# Winning the Genetic Lottery: Biasing Birth Sex Ratio Results in More Grandchildren

**DOI:** 10.1371/journal.pone.0067867

**Published:** 2013-07-10

**Authors:** Collette M. Thogerson, Colleen M. Brady, Richard D. Howard, Georgia J. Mason, Edmond A. Pajor, Greg A. Vicino, Joseph P. Garner

**Affiliations:** 1 Ecological Services, U.S. Fish & Wildlife Service, Arlington, Virginia, United States of America; 2 Department of Animal Sciences, Purdue University, West Lafayette, Indiana, United States of America; 3 Department of Biological Sciences, Purdue University, West Lafayette, Indiana, United States of America; 4 Department of Animal and Poultry Science, University of Guelph, Ontario, Canada; 5 Veterinary Medicine, University of Calgary, Alberta, Canada; 6 Collections Husbandry Science, San Diego Zoo Global, San Diego, California, United States of America; 7 Department of Comparative Medicine, and by Courtesy, Department of Psychiatry and Behavioral Sciences, Stanford University, Stanford, California, United States of America; University of Turku, Finland

## Abstract

Population dynamics predicts that on average parents should invest equally in male and female offspring; similarly, the physiology of mammalian sex determination is supposedly stochastic, producing equal numbers of sons and daughters. However, a high quality parent can maximize fitness by biasing their birth sex ratio (SR) to the sex with the greatest potential to disproportionately outperform peers. All SR manipulation theories share a fundamental prediction: grandparents who bias birth SR should produce more grandoffspring via the favored sex. The celebrated examples of biased birth SRs in nature consistent with SR manipulation theories provide compelling circumstantial evidence. However, this prediction has never been directly tested in mammals, primarily because the complete three-generation pedigrees needed to test whether individual favored offspring produce more grandoffspring for the biasing grandparent are essentially impossible to obtain in nature. Three-generation pedigrees were constructed using 90 years of captive breeding records from 198 mammalian species. Male and female grandparents consistently biased their birth SR toward the sex that maximized second-generation success. The most strongly male-biased granddams and grandsires produced respectively 29% and 25% more grandoffspring than non-skewing conspecifics. The sons of the most male-biasing granddams were 2.7 times as fecund as those of granddams with a 50∶50 bias (similar results are seen in grandsires). Daughters of the strongest female-biasing granddams were 1.2 times as fecund as those of non-biasing females (this effect is not seen in grandsires). To our knowledge, these results are the first formal test of the hypothesis that birth SR manipulation is adaptive in mammals in terms of grandchildren produced, showing that SR manipulation can explain biased birth SR in general across mammalian species. These findings also have practical implications: parental control of birth SR has the potential to accelerate genetic loss and risk of extinction within captive populations of endangered species.

## Introduction

Sex ratio (SR) manipulation theory is one of the founding pillars of sociobiology and modern evolutionary theory [1]–[3]. Early work on frequency-dependent selection on gender and resulting population dynamics (notably Fisher [1]) is celebrated for showing that advantages to individual parents will lead to equal investment, and stabilize the birth population sex ratio at 50∶50. Sex allocation theory, first proposed by Hamilton [2], builds on and also challenges this work. If offspring sex can be manipulated, and a grandparent can predict the likely success of their offspring, then a grandparent can obtain a fitness advantage (in terms of grandchildren produced) by biasing its birth sex ratio (SR) in favor of the sex with the greatest potential to disproportionately outperform peers, disproportionately contribute to inclusive fitness, or fail to compete the least [2]. The physiology of mammalian sex determination is supposedly stochastic, producing equal numbers of sons and daughters. Nevertheless if functional consequences of SR manipulation were to be found in mammals, then it would suggest that mammals (either in individual species, or in general), possess unknown physiological mechanisms to control birth SR. Such a bridge between evolutionary and basic molecular biology would be one of the most exciting implications of SR manipulation (e.g. [4]).

Hamilton [2], focused on scenarios specific to particular insect groups. In Mammals and birds a more general principle applies: the number of offspring a male produces is often limited by how many females he can mate with, while a female is limited by how many offspring she can physiologically produce [5], [6].This generates a tendency for males to vary more in first-generation success than females. Thus male offspring are a high-risk-high-reward bet for potential grandparents in the genetic lottery; while females are a safe, hedged bet [5], [6]. However, just like in insects, if a grandparent ‘knows’ that a male offspring is a low-risk-high-reward bet, then they can beat the house, and hit a jackpot (in terms of grandchildren produced) [5], [6]. Furthermore, grandparents can beat the house in other more subtle ways, leading later authors to propose a variety of advantages to SR manipulation that might apply to vertebrate species (e.g. local resource competition or enhancement [3]). Each of these different SR manipulation theories proposes different corresponding cues that grandparents might use to predict the success of their offspring, and corresponding selective pressures underlying these benefits (for an excellent review, see [3]). For example (and most obviously), parents can produce more of the sex most likely to out-reproduce peers [2]; and either grandparent’s quality or social status may be excellent cues for the subsequent reproductive success of their offspring relative to their potential competitors [5], [6]. Thus high quality granddams may bias towards males. Conversely, a low quality or stressed granddam, may bias towards daughters, not because they will outcompete peers, but because their failure to compete will be less impactful than that of a son. Although this example (the “Trivers-Willard Hypothesis”) is the most famous, other benefits clearly occur through biasing towards the sex which can reduce reproductive costs or competition, or maximize inclusive fitness (for instance via enhanced production of the sex that disperses; or the sex that provides care for younger siblings, respectively [7]). Similarly, simple sexual selection can drive bias – for instance, a granddam should bias towards males if the grandsire excels in a sexually selected heritable trait that will result in ‘sexy sons’, enhanced sperm competition, or other reproductive advantages distinct from the maternal quality emphasized by the Trivers-Willard Hypothesis. In nature, mammalian parents often do bias birth SR in correlation with physiological, behavioral, or environmental cues that are in turn consistent with these ideas (e.g. [8]–[10]). For instance, dominant red deer mothers skew their SRs toward sons, which is tantalizing as red deer stags with greater mating success tend to have mothers of higher dominance [6].

However, while this and other examples suggest that SR manipulation could be adaptive, and are often taken as evidence of such, they in fact provide only circumstantial evidence [3]. This example, and all other mammalian studies to our knowledge, require a leap of faith – the true test is to demonstrate that grandparents with skewed birth SRs produce more grandchildren than their peers [3], and that this benefit accrues specifically through the biased individuals in the intermediate generation. In other words, if a grandmother biases towards sons (for example), then those particular sons must outcompete other males in their generation to produce her more grandchildren in total, and more grandchildren per son. Thus all SR manipulation theories (from Trivers-Willard, to sexy sons, to local resource competition or enhancement) all ultimately make the same prediction: that favoring the sex with the greatest potential to disproportionately outperform peers, disproportionately contribute to inclusive fitness, or fail to compete the least, will mean that biased F1 individuals should produce more F2 offspring *per capita* than their non-biased peers. The power of this prediction is that it is agnostic to the particular theory under test, the particular cues grandparents may be responding to, or the direction of bias; and hence should be general across mammals irrespective of mating systems, natural history, or their particular responses to captivity. However, it has an Achilles’ heel – testing it requires a complete three-generation pedigree where every grandchild of every grandparent is known [3], [11], which is practically unobtainable in the wild. Thus Clutton-Brock’s seminal work in red deer [6] could not test whether the females that produced more sons actually gained more grandchildren; nor whether the successful sons descended from the particular females who biased (because not all the dominant females did actually bias). Instead they could only show that dominant females produce more sons; and that males with more offspring had more dominant mothers. Thus, the most successful males could just as easily have come from the dominant dams who only invest in one ‘super son’ offspring (which would falsify the hypothesis). As a result, the empirical work in SR manipulation has come under increasing criticism in recent years (e.g. [3], [11]), not least because other predicted effects have been much more elusive. In particular, SR theory predicts that males should also control birth SR [2] (and arguably the mechanisms are far more straightforward for them to do so in mammals [12]), but to date examples have been very rare [12], [13].

Given the power of SR manipulation’s theoretical argument, the compelling but circumstantial field data in the literature, and the implications for basic reproductive physiology; our goal was to test the central, yet untested, predictions of SR manipulation theory – that skewing birth SRs enhances parental fitness and that offspring of the favored sex out-reproduce their peers. To do so required overcoming the hurdle of obtaining the three-generation pedigree required. Our solution was to use 90 years of breeding records from San Diego Zoo Global (SDZG) to reconstruct the complete three-generation pedigrees for 198 species of *Artiodactyla*, *Perissodactyla*, *Carnivora*, and *Primates.* Grandmothers and Grandsires who biased their birth SR gained more grandchildren – specifically via disproportionate success of the individual favored offspring. To our knowledge this is the first demonstration of the key prediction of SR manipulation theory in mammals, vindicating the earlier classic field studies that could not build the three-generation pedigrees required.

## Materials and Methods

### Ethics Statement

Prior to data collection, we confirmed with Purdue University’s Institutional Animal Care and Use Committee that no ethical approval was required for this kind of study; data was collected using historical records.

### Source Records, Subjects, Exclusion criteria, and Data Processing

Using breeding records from San Diego Zoo Global (SDZG), we compiled data on 38,075 individuals from 678 mammalian species spanning the *Artiodactyla*, *Perissodactyla*, *Carnivora*, and *Primates*. We constructed pedigrees for grandparents (the F0 generation) only including those for whom we could follow the breeding of all their children (the F1 generation, totaling 11,909 for granddams; 11,563 for grandsires) to calculate the F0’s success as the number of grandchildren produced (the F2 generation, totaling 16,553 for granddams, 12,895 for grandsires). This measure of success lets us test the most global prediction of SR manipulation theory – that F0 individuals which have biased birth SR will have more grandchildren. We also calculated F0 success as the grandchildren (F2) born per each of their reproductive F1 offspring; which explicitly examines success via the biased sex, and accounts for those F1 individuals that do not breed, and tests the most explicit prediction of SR manipulation – that F0s who bias increase F2 success through F1 offspring who outperform their peers. This resulted in pedigrees for 1627 granddams and 703 grandsires. F1 offspring were counted for each grandparent, to calculate their lifetime birth SR. These birth SRs were corrected for the role of chance (a 100% male birth SR is much more impressive given six offspring than three) by expressing them as *Z-*scores, following classic experimental work on birth SR [6]. The *Z-*score, or normal approximation to the binomial, calculated from observed and expected proportions, is given by [14] (and given Fisher’s argument for a population level zygotic SR of 50∶50, setting the expected birth SR = 0.5):
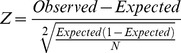



We calculated the lifetime birth SR, not the per-litter SR to allow for a fair comparison between the monotocous and polytocous species in the data set. A per-litter SR would be dichotomous for monotocous species, but continuous for polytocous species. The lifetime SR avoids this confound, but means that we assume that females’ mean lifetime SR is meaningful. This is a conservative assumption: the analysis will pick up females who consistently bias across their lives, but will miss out on the litter-level information from females who might have a one-off opportunity to produce a single litter with highly successful sons (or daughters). Thus, as with all of our analytical choices, we opted for the risk of a false negative, rather than the risk of a false positive.

### Statistical Methods

Blocking by Species nested within Order, F0 animals’ *Z-*scored birth SRs were regressed against the total number of F2 offspring (total grandchildren); and also, the average number of F2 grandoffspring produced by each F1 offspring of the favored sex. Including Species in the analysis identifies F0 individuals as belonging to the same species, which avoids pseudoreplication, and also ensures that the results for *Z*-scored birth SR represent the mean within-species regression. Analyses were repeated with controls for human management namely: blocking for the year in which each F0 subject first bred and its interaction with Order (since SDZG breeding regimes changed over the decades); and for the proportion of F1 offspring bred (which would reflect the perceived genetic value of a grandparent). All analyses yielded the same pattern of results. Therefore, we present the most conservative analyses including all controls for management. All analyses were initially performed including interactions of *Z*-score and Order, to test for different mean relationships in the different taxa. However none of these interactions were significant, and were therefore removed from the final analysis to ensure marginality [15]. For granddams, 44 of 193 species were represented by a single female; and for grandsires, 67 of 197 species were represented by a single male. These data points were inherently excluded by the analysis, as it tested for within-species effects (final distributions are provided in Materials S1). All analyses were performed as GLMs in JMP 9.0 for Windows. The assumptions of GLM (normality of error, homogeneity of variance, and linearity) were confirmed *post hoc* and suitable transformations applied as needed [15].

## Results

**Figure 1:**
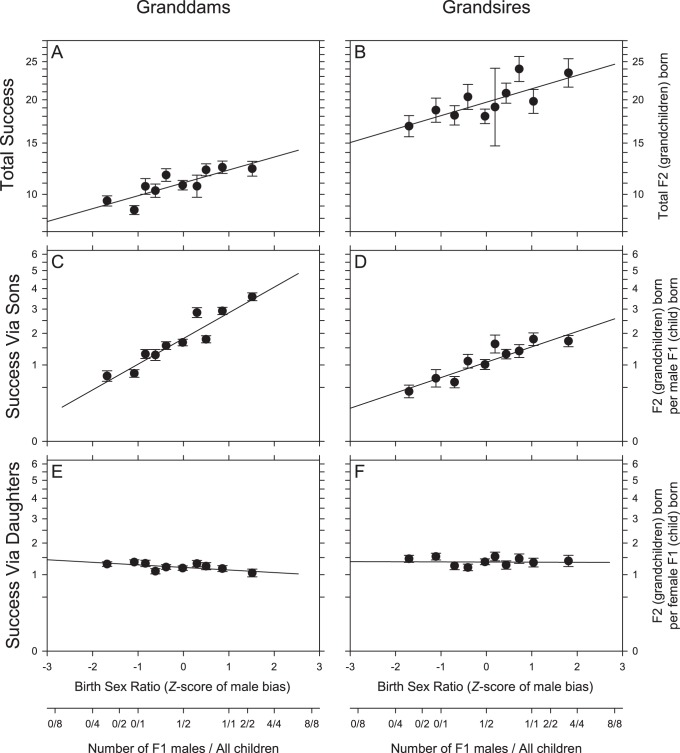
Grandparents who bias the sex of the offspring, have more successful offspring, gaining more grandchildren. **A**) Granddams and **B**) grandsires who biased birth SR towards males had greater total success measured as total grandchildren produced (*P*<0.0001; *P = *0.0108, respectively). Birth SR is shown as a *Z-*score, to control for number of F1 offspring (the X-axes also give examples of male biases for a given *Z-*score). **C**) Granddams, and **D**) grandsires, who biased birth SR towards males had greater success specifically via F1 males (for both, *P*<0.0001). **E**) Granddams who biased birth SR towards females had greater success specifically via F1 females (*P = *0.0272), but no effects were found for female-biasing grandsires (*P* = 0.9426), (nor did they have more total grandchildren overall; see text). For clearer data visualization, the data were split into 10th percentiles by *Z-*score, and plotted values are least-squares means and standard errors within those percentiles. The solid line indicates the least-squares regression line partialled for the controlling variables. In **A** and **B**, the Y-axes shows F0 success as total grandchildren born. In **C**–**F**, granddam and grandsire success is shown as the grandchildren (F2) born per each of their F1offspring born of a given sex (i.e. the mean reproductive output of the F1 children of each sex).

F1 population-level birth SRs proved slightly female biased (for granddams 47.5% of F1 offspring were male). Birth SR varied greatly across F0 subjects (86.8% of variance in SR occurred within-species for granddams, and 72.4%for grandsires; see Table 1 for particularly variable species) and this variation did indeed have adaptive consequences in general across the range of species in the data set. Granddams who biased their birth SR towards sons gained more grandoffspring in total (GLM: *F*_1,1427_ = 26.45: *P*<0.0001; *N* = 1627 granddams; **Figure 1A**), as did grandsires who male-biased their birth SR (GLM: *F*_1,499_ = 6.553: *P*<0.0108; *N* = 703 grandsires; **Figure 1B**). The most strongly male-biased granddams and grandsires produced respectively 29% and 25% more grandoffspring than non-skewing conspecifics. The more male-biased a granddam’s birth SR, the more grandoffspring she gained from each son (GLM: *F*_1,1260_ = 194.9: *P*<0.0001; *N* = 1454 granddams; **Figure 1C**): the sons of the most male-biasing granddams were 2.7 times as fecund as those of granddams with a 50∶50 bias. Similar effects held for grandsires (GLM: *F*_1,468_ = 27.53: *P*<0.0001; *N* = 678 grandsires; **Figure 1D**). The more female-biased a granddam’s birth SR, the more grandoffspring she gained from each daughter (GLM: *F*_1,1390_ = 4.891: *P* = 0.0272; *N* = 1558 granddams; **Figure 1E**); effects were smaller however – daughters of the strongest F0 female-biasing females were 1.2 times as fecund as those of non-biasing females. The SR bias of grandsires had no effect on the number of grandchildren gained from each daughter. (GLM: *F*_1,476_ = 0.0052: *P* = 0.9426; *N* = 678 grandsires; **Figure 1F**).

**Table 1:** Species with notably skewed Birth Sex Ratios.

Common name	Scientific name	Variance in *Z*-score SR	*N*	*F*	*P*
***Granddams***					
Vaal Rhebok	*Pelea capreolus*	3.089	4	3.560	0.0138
Sumatran Tiger	*Panthera tigris*	4.197	2	4.838	0.0280
Red River Hog	*Potamochoerus porcus*	2.320	5	2.674	0.0306
Sudan Red-fronted Gazelle	*Gazella rufifrons*	2.061	7	2.376	0.0274
Gambian Maxwell’s Duiker	*Cephalophus maxwellii*	2.231	5	2.572	0.0363
***Grandsires***					
Kenya Impala	*Aepyceros melampus*	4.880	5	4.777	0.0009
Indochinese Sika	*Cervus Nippon*	2.528	6	2.475	0.0314
Francois’ Langur	*Trachypithecus francoisi*	2.821	4	2.761	0.0416
East African Black Rhinoceros	*Diceros bicornis*	4.000	2	3.916	0.0484
Nubian Ibex	*Capra nubiana*	2.024	8	1.982	0.0558

The variance in birth SR was figured for each species. The five species with the greatest variance (i.e. standard deviation^2^) in F1 birth SR for granddams and grandsires are listed. Because birth SR is expressed as *Z-*score, the expected variance for any species = 1. The observed variances are tested against the mean within-species variance in *Z-*score.

## Discussion

These data clearly demonstrate the ultimate reason why parents control birth SR: parents who are able to judge the future success of their first generation offspring and bias their birth SR accordingly have a clear F2 fitness advantage over those who cannot. Therefore, as first clearly postulated over 40 years ago [2], but to our knowledge demonstrated here for the first time, sex ratio manipulation is a widespread and highly adaptive evolutionary strategy in mammals. The most global prediction of SR manipulation theory – that individuals who manipulate their birth SR will have more grandchildren through the improved reproductive output of F1 offspring of the biased sex – was thus supported. Specifically, the sons of granddams with male-biased birth SRs out-perform their peers, yielding these granddams more grandchildren. The same holds for grandsires. Furthermore these data, to our knowledge, show for the first time that the daughters of female-biasing granddams likewise outperform their peers. The differential effects seen in the analyses of success via the F1 sex are consistent with existing understanding of factors influencing birth SR in granddams [5]–[7], [13], [16]–[22]. Thus, the lack of influence of grandsire BSR on F2 success via F1 daughters was expected, and the very weak effect of granddam BSR on F2 success via F1 daughters likely reflects a subset of ‘special-case’ species, rather than a general effect.

The use of captive populations warrants comment, as it is a double-edged sword. Because the populations are captive, and because the provenance of individual animals is so critical in captive breeding, we can reconstruct pedigrees across a breadth of species and to a depth of generations that would be impossible in the wild. Indeed, the previous lack of a strong test of SR manipulation in terms of grandchildren is itself compelling evidence for just how difficult it is to produce a complete three-generation pedigree in a wild mammalian population. Such a data set is obviously not impossible, but it is sufficiently impractical to have eluded researchers for forty years. Thus using data from captive animals is not so much of an advantage, but currently a necessity. Zoo populations represent the best choice for a sample population compared to farm or laboratory animals, because they provide a breadth of species, and they enjoy a more naturalistic environment. This last point however, reveals the downside of working with zoo populations – that they are still managed populations where breeding is to a degree under human control, and where the environment may differ from captivity in ways that present misleading cues to animals in terms of SR manipulation. The level of management, and the qualitative match of the captive to the wild environment, both differ in particular by taxon and between modern *versus* historical populations. For instance, many hoofstock species at SDZG live in a relatively free ranging herd and males may experience direct competition resulting in female mate choice; while primate species may not be provided rival mates simultaneously. However, even in the absence of perceived mate choice, females can often control whether or not they mate, the chance of conception, and *in utero* and post natal investment in offspring from different males. Most importantly, however, captive breeding programs track the provenance of potential mates and explicitly attempt to minimize the rate of loss of genetic diversity caused by population bottlenecks (e.g. by limiting the number of mating opportunities given to particular sires). However, for our analyses, such effects should–if anything–produce false negatives; and they certainly could not produce false positives. Thus a misleading environmental cue might cause females to bias their SR, but the resulting biased offspring would not benefit. Similarly, if humans are controlling breeding to maximize genetic diversity then they will curtail the success of high quality males, and boost the success of low quality males. Our results have thus emerged despite a strong potential for adaptive SR effects to be masked in captivity. They are thus extremely conservative, and suggest that were it possible to follow three generations or more in the wild, even stronger effects would be evident. Consequently, the analyses are explicitly designed to capitalize on the strengths of these data, while protecting against the potential weaknesses. Thus, by testing for general birth SR trends across the controlling variables, the analysis exploits the heterogeneity of the data (in terms of species, year of breeding, husbandry systems, *etc.*) to ensure that any result is general, rather than a specific artifact driven by a particular species or husbandry system. Similarly, by testing for specific predictions via the F1 genders separately, the analyses guard against a general false positive tainting the whole data set. Finally, by testing for specific benefits in F2 success to the F0 grandparents, deriving from an improved success of the biased F1 offspring, we test the common prediction of all SR manipulation theories, and are therefore agnostic as to which of the cues or mechanisms (e.g. Trivers-Willard, or Local Resource Enhancement), might be responsible.

Overall, despite a dataset where such effects could be masked by human attempts to control breeding, skewing birth SR is confirmed (thanks to unprecedented sample sizes and complete, accurate counts of grandoffspring) as a widely utilized strategy across captive *Mammalia* to enhance maternal and paternal fitness, but is likely extended to wild *Mammalia*. Furthermore, our findings newly identify ideal species (those with high variances in birth SR: see Table 1) for future research on proximate mechanisms underlying mammalian SR manipulation, the physiology of which remains unknown [3], [10], [11]. These findings have additional practical implications too: in captive populations under human control, these individually adaptive strategies may be significantly impacting the long term genetic viability of the species and compromising the captive population as a whole. The increased variability in F2 success between grandsires *versus* granddams apparent in Figures 1A –1D, neatly illustrates the species-level cost of SRM in small populations. Winners of the genetic lottery do so at others’ expense, and a highly successful F1 male over-contributes to the next generation at the species-level cost of the loss of genetic variation from the males that fail to breed. Thus the general tendency of captive species to bias their birth SR demonstrated here (especially in F1 males), combined with the fact that many captive species have male biased BSR [23], [24], has the potential to accelerate the loss of genetic diversity from endangered captive-bred species. Accordingly understanding the factors leading to biased birth SR in captive populations, and thus identifying potential interventions to manipulate birth SR, will be critical to the effective preservation of genetic diversity in captive breeding.

## Supporting Information

Materials S1 (DOCX)
