# Supplementary material for: Winning the Genetic Lottery: Biasing Birth Sex Ratio Results in More Grandchildren
Source: PLoS One. 2013 Jul 10;8(7):e67867. doi: 10.1371/journal.pone.0067867 (PMC3707872; doi:10.1371/journal.pone.0067867)
Supplement: Materials S1 — (DOCX) [file pone.0067867.s001.docx]

# Supplementary materials to Thogerson *et al*., “Winning the Genetic Lottery: Biasing Birth Sex Ratio Results in More Grandchildren”

The raw data was reduced as described in the methods to yield three-generation pedigrees. F0 (grandparents) were excluded from the data if either their F1 or F2 offspring could not be fully accounted for, yielding 1627 granddams and 703 grandsires. For granddams, 44 of 193 species were represented by a single F0 female; and for grandsires, 67 of 197 species were represented by a single F0 male. These data points were included so that the mean fecundity for each Order was estimated as accurately as possible, but excluded from the estimates and test for *Z*-Score by the analysis itself, as it explicitly tested for within-species effects.

The Tables and Figures below show the distribution of the number of F0 individuals per species for grandams and grandsires.

# Number of Granddams per Species

**Distribution**

**Quantiles**

|  |  |  |
| --- | --- | --- |
| 100.0% | maximum | 51 |
| 99.5% |  | 51 |
| 97.5% |  | 35.15 |
| 90.0% |  | 21 |
| 75.0% | quartile | 12 |
| 50.0% | median | 5 |
| 25.0% | quartile | 2 |
| 10.0% |  | 1 |
| 2.5% |  | 1 |
| 0.5% |  | 1 |
| 0.0% | minimum | 1 |

**Summary Statistics**

|  |  |
| --- | --- |
| Mean | 8.4300518 |
| Std Dev | 9.4536439 |
| Std Err Mean | 0.6804882 |
| Upper 95% Mean | 9.7722443 |
| Lower 95% Mean | 7.0878593 |
| N | 193 |

# Number of Grandsires per Species

**Distribution**

**Quantiles**

|  |  |  |
| --- | --- | --- |
| 100.0% | maximum | 27 |
| 99.5% |  | 27 |
| 97.5% |  | 11 |
| 90.0% |  | 7.2 |
| 75.0% | quartile | 5 |
| 50.0% | median | 3 |
| 25.0% | quartile | 1 |
| 10.0% |  | 1 |
| 2.5% |  | 1 |
| 0.5% |  | 1 |
| 0.0% | minimum | 1 |

**Summary Statistics**

|  |  |
| --- | --- |
| Mean | 3.5685279 |
| Std Dev | 3.2610291 |
| Std Err Mean | 0.2323387 |
| Upper 95% Mean | 4.0267326 |
| Lower 95% Mean | 3.1103232 |
| N | 197 |
